# Porcine Epidemic Diarrhea Virus (PEDV) ORF3 Enhances Viral Proliferation by Inhibiting Apoptosis of Infected Cells

**DOI:** 10.3390/v12020214

**Published:** 2020-02-14

**Authors:** Fusheng Si, Xiaoxia Hu, Chenyang Wang, Bingqing Chen, Ruiyang Wang, Shijuan Dong, Ruisong Yu, Zhen Li

**Affiliations:** 1 Institute of Animal Science and Veterinary Medicine, Shanghai Key Laboratory of Agricultural Genetics and Breeding, Shanghai Academy of Agricultural Sciences, Shanghai 201106, China; mr.fusheng@163.com (F.S.); 13122257810@163.com (X.H.); chenyang_1wang@163.com (C.W.); c13919966733@163.com (B.C.); ruiy1220@163.com (R.W.); dsjnm@163.com (S.D.); yursong@163.com (R.Y.); 2 Shanghai Engineering Research Center of Breeding Pig, Shanghai 201106, China; 3 National Demonstration Center for Experimental Fisheries Science Education, Shanghai Ocean University, Shanghai 201306, China

**Keywords:** PEDV, the role of ORF3, proliferation, apoptosis, cells

## Abstract

The genomes of coronaviruses carry accessory genes known to be associated with viral virulence. The single accessory gene of porcine epidemic diarrhea virus (PEDV), ORF3, is dispensable for virus replication in vitro, while viral mutants carrying ORF3 truncations exhibit an attenuated phenotype of which the underlying mechanism is unknown. Here, we studied the effect of ORF3 deletion on the proliferation of PEDV in Vero cells. To this end, four recombinant porcine epidemic diarrhea viruses (PEDVs) were rescued using targeted RNA recombination, three carrying the full-length ORF3 gene from different PEDV strains, and one from which the ORF3 gene had been deleted entirely. Our results showed that PEDVs with intact or naturally truncated ORF3 replicated to significantly higher titers than PEDV without an ORF3. Further characterization revealed that the extent of apoptosis induced by PEDV infection was significantly lower with the viruses carrying an intact or C-terminally truncated ORF3 than with the virus lacking ORF3, indicating that the ORF3 protein as well as its truncated form interfered with the apoptosis process. Collectively, we conclude that PEDV ORF3 protein promotes virus proliferation by inhibiting cell apoptosis caused by virus infection. Our findings provide important insight into the role of ORF3 protein in the pathogenicity of PEDV.

## 1. Introduction

Porcine epidemic diarrhea (PED) and its causative pathogen, porcine epidemic diarrhea virus (PEDV), were first recognized in Europe in the 1970s [1,2]. The disease spread to Asia in the 1980s and caused great economic losses to the pig industry in the area, especially after 2010 [3,4,5]. In 2013, the disease also emerged and spread rapidly in North America, caused by a virus that most likely originated from Asia [6].

PEDV is a positive-sense RNA virus classified in the genus *Alphacoronavirus*, family *Coronaviridae*. Its genome, about 28 kb in length, encodes two nonstructural polyproteins (pp1a and pp1ab), four structural proteins—the approximately 200 kDa glycosylated spike (S) protein, 8 kDa envelope (E) protein, 24 kDa membrane (M) protein, and 58 kDa nucleocapsid (N) protein—and one known accessory protein, the ORF3 protein [7,8]. Pp1a and pp1ab are the precursors of several enzymes and cofactors that together carry out PEDV genome replication and transcription. The S glycoprotein, trimers of which protrude from the viral membrane providing the coronaviruses their typical appearance, functions during cell entry by binding to cellular receptors and causing fusion of the viral and host cell membranes. The E protein has ion channel activity [9] and plays an important role in virion morphogenesis. The M protein is the main component of the viral envelope and interacts with all structural components during viral particle assembly. The N protein packages the genomic RNA to form the helical nucleocapsid (RNP). 

ORF3 is located between the S and E genes; it is the only conserved ORF in all the genus of coronaviruses except *Deltacoronavirus* [10]. PEDV ORF3 encodes a protein of 224 residues, which is about 25 kDa, but the gene is prone to undergo a 49–51 nucleotide (nt) deletion mutation when the virus is adapted to growth in cell culture, e.g., by serial passaging [11,12]. The 49-nt deletion leads to a premature translation stop at nt 274 giving rise to a naturally truncated ORF3 protein of 92 residues designated ORF3^trun^, as is the case with ORF3 of the attenuated DR13 strain (DR13^att^), which was used as the backbone of the recombinant viruses generated in the present study. Another type of ORF3 variant, as occurring in PEDV strains 85-7 and AVCT12, has a 30 nt deletion at the 5′-end giving rise to a truncated ORF3 protein missing 70 residues at its N-terminus [13,14]. Field isolates with longer-length deletions of ORF3 have also been documented [15]. 

While there is accumulating evidence showing the ORF3 protein to be related to PEDV pathogenicity, the underlying mechanism is still elusive. The protein has been reported to function as an ion channel in both Xenopus laevis oocytes and yeast cells. Suppression of ORF3 expression by siRNA was found to inhibit the production of wild-type PEDV but not that of attenuated-type PEDV [16]. Using a Vero cell line stably expressing ORF3, it was demonstrated that PEDV ORF3 protein prolonged the S phase of the cell cycle besides augmenting vesicle formation in the cells. Interestingly, constitutive expression of the ORF3 protein exerted a positive regulatory effect on the proliferation of attenuated PEDV but not on that of virulent PEDV [17]. In a more recent study, both the wild type and a mutant ORF3 protein lacking residues 82–98 were found to co-localize with the S protein intracellularly and at the cell surface, both in infected cells and during co-expression in transfected cells [18]. Additionally, a direct interaction of the S protein with each of these ORF3 proteins was demonstrated in such transfected cells by co-immunoprecipitation, leading the authors to suggest that the ORF3 protein might be involved in virus assembly. 

To extend our knowledge about the biological role of the ORF3 protein in PEDV infection, the aim of the present study was to reveal its effect on viral replication and to gain further insight into its role in pathogenicity. We generated four isogenic recombinant porcine epidemic diarrhea viruses (rPEDVs) based on the genomic backbone of strain DR13^att^, three of them carrying an intact ORF3 derived from different virus strains and one from which ORF3 had been entirely deleted. With these rPEDVs and DR13^att^, we studied the function of ORF3 by comparing the titers of the viruses and the biological characteristics of the infected cells. We found that the ORF3 protein enhanced the proliferation of PEDV by a mechanism most likely involving inhibition of apoptosis in infected cells. 

## 2. Materials and Methods 

### 2.1. Cells, Viruses, and Antibodies

Murine L (LR7) cells (a L-2 murine fibroblast cell line stably expressing the murine hepatitis virus receptor, a gift of Peter Rottier, Utrecht University) and Vero CCL-81 cells (African green monkey kidney cells, purchased from ATCC) were cultured in Dulbecco’s modified Eagle’s medium (DMEM; Gibco, Invitrogen, Carlsbad, CA, USA) supplemented with 10% fetal bovine serum (FBS; Gibco, Invitrogen, Carlsbad, CA, USA), penicillin (100 U/mL), and streptomycin (100 μg/mL) at 37 °C in a humidified atmosphere with 5% CO_2_. The cell culture-adapted DR13^att^ (JQ023162; isolated from a commercial vaccine of GreenCross, South Korea), the rPEDVs, respectively, carrying the ORF3 of wild-type PEDV DR13 (JQ023161; rDR13^att^-ORF3^wt^), of wild-type CV777 (AF353511; rDR13^att^-ORF3^CV777^), and of local PEDV field strain NY (rDR13^att^-ORF3^NY^), and a recombinant PEDV without ORF3 (rDR13^att^-∆ORF3) were propagated and titrated in Vero cells (for the sequences of these ORF3s and their encoded proteins, see Figure S1). 

Rabbit anti-PEDV S polyclonal antibody was produced against a bacterially expressed C-terminal 423-aa domain of the spike protein (residues 961-1383) derived from the CV777 strain (accession No. AF353511). Rabbit anti-ORF3 polyclonal antibody (P71-3) was produced against a synthetic N-terminal 12-aa peptide (^10^IDTVVKDVSKSA^21^) of the ORF3 protein (ORF3^wt^) of PEDV DR13 (JQ023161). Mouse anti-PEDV N protein monoclonal antibody 3F.12 (Catalog No. 9191) was purchased from BioNote, Hwaseong-si, South Korea. HRP-labeled goat anti-rabbit IgG (Catalog No. D110058), HRP-labeled goat anti-mouse IgG (Catalog No. D110087), and rabbit anti-GAPDH polyclonal antibody (Catalog No. D110016) were purchased from Sangon Biotech Inc., Shanghai China. Alexa Fluor 488 conjugated goat anti-mouse IgG (Catalog No. A0428), Alexa Fluor 488 conjugate goat anti-rabbit IgG (Catalog No. A0423) and Alexa Fluor 647 conjugated goat anti-mouse IgG (Catalog No. A0473) were purchased from Beyotime Biotech Inc., Shanghai China. Rabbit anti-cleaved caspase-3 monoclonal antibody (Catalog No. 9664) and rabbit anti-caspase-3 monoclonal antibody (Catalog No. 9665) were purchased from Cell Signaling Technology, Beverly, MA, USA. Alexa Fluor 594 conjugate goat anti-rabbit IgG (Catalog No. R37117) was purchased from ThermoFisher, Carlsbad, CA, USA. PE-conjugated monkey anti-rabbit secondary antibody (Catalog No. 12-4739-81) and FITC-conjugated rat anti-mouse secondary antibody (Catalog No. 11-4011-85) were purchased from eBioscience (San Diego, CA, USA).

### 2.2. Synthesis of Full Length ORF3^wt^ cDNA of Parental PEDV DR13

A full-size 675 bp ORF3 cDNA of the wild-type PEDV strain DR13 (JQ023161) was obtained from overlapping synthetic cDNA fragments by PCR [19]. 

### 2.3. Amplification of ORF3^CV777^ and ORF3^NY^ Gene

RNA of PEDV CV777 and PEDV^NY^ were isolated from a culture supernatant of PEDV infected Vero cells and from a PEDV-positive fecal specimen collected from a pig farm located in the Nanyang suburb (Henan, China), respectively, by using AxyPrep and TRIzol kits (Invitrogen, Carlsbad, CA, USA). cDNA was prepared by using primer 2 (Table 1) and the extracted RNAs as a template. The ORF3^CV777^ and ORF3^NY^ genes were amplified from their cDNA in a PCR reaction mixture containing: 1 μL each of primers 1 and 2 (Table 1; 10 μM each), 2 μL cDNA, 5µL reaction buffer, 3 µL dNTP mix (10 µM each), 1 µL *PrimeSTAR HS* DNA polymerase (Takara, Japan), and 14 µL pure water. The resulting PCR products were harvested, cloned into pJET 1.2 blunt vector, and designated pJET 1.2-ORF3^CV777^ and pJET 1.2-ORF3 ^NY^ after verification by sequencing.

### 2.4. Recombinant Plasmid Constructions

Construction of vectors pPEDV-DR13^att^, pDR13^att^-ORF3^wt,^ and pDR13^att^-∆ORF3 has been described previously [20]. 

To construct pDR13^att^-ORF3^CV777^ and pDR13^att^-ORF3^NY^, DNA fragments comprising the ORF3 gene were cut out from pJET 1.2-ORF3^NY^ and pJET 1.2-ORF3^CV777^ with *Pml*I and *Age*I and then ligated into pPEDV-DR13^att^ digested with the same enzymes, respectively. The acquired recombinant plasmids were verified by sequencing.

### 2.5. Rescue and Identification of the Recombinant PEDVs

Rescue of the recombinant PEDVs was carried out as described previously [20]. Briefly, LR7 cells were grown to 90% confluence and infected with mPEDV (a recombinant PEDV in which the S gene was substituted by that of a mouse hepatitis virus, which enabled the recombinant virus to infect murine LR7 cells rather than Vero cells) at a multiplicity of infection (MOI) of 1.0. When obvious cytopathic effect (CPE) appeared, cells were trypsinized to produce a single cell suspension and washed 3 times with PBS. Capped runoff RNA transcripts (donor RNA) were synthesized from *Pac*I-linearized pDR13^att^-∆ORF3, pDR13^att^-ORF3^wt^, pDR13^att^-ORF3^CV777^, and pDR13^att^-ORF3^NY^, respectively, using a T7 RNA polymerase kit (Ambion, Carlsbad, CA, USA), as specified by the manufacturer. Then the donor RNAs were transfected into the above mPEDV infected LR7 cells by electroporation (300 V, 975 μF, 2 consecutive pulses) using a Gene Pulser apparatus (SCIENTZ-2C, Ningbo Scientz Biotech Co, Ltd, Ningbo, China). Finally, the electroporated cells were resuspended in DMEM supplemented with 2% FBS and co-cultured in a 25 cm^2^ flask with a monolayer of Vero cells. After 4–5 days of incubation at 37 °C, the cultures were harvested by 3 cycles of freeze-thawing and candidate recombinant viruses were purified by 3 rounds of end-point dilutions on Vero cells. Finally, virus stocks were grown in Vero cells, titrated, and stored at −80 °C. The viral RNAs were then extracted with Trizol reagent (Invitrogen, Carlsbad, CA, USA) and cDNAs were synthesized using primer 2 (Table 1) and the extracted RNA. RT-PCR was performed by using primers 1 and 2 (Table 1) and TransScript One-Step RT-PCR SuperMix (TransGen Biotech Inc., Beijing, China). The amplified genes were sequenced and assembled using the MEGA 6.0 software. For identification of rDR13^att^-∆ORF3, cDNA was synthesized with primer 2 (Table 1) and RT-PCR was performed by using primers 4 and 5 to amplify the S-E-∆ORF3 fragment for sequencing.

### 2.6. Virus Titration and Growth Curve Determination

Viral titers were measured in 96-well tissue culture plates (ThermoFisher, Carlsbad, CA, USA) using 50% tissue culture infective dose (TCID_50_) assays according to the procedure described previously [19].

For the virus growth curve determination, Vero cells in 6-well plates were inoculated with the rescued recombinant viruses or with control viruses at an MOI of 0.1. After adsorption at 37 °C for 60 min, the cells were fed with the maintenance medium, and incubated at 37 °C under 5% CO_2_. At 8, 16, 24, 32, 40, and 48 h.p.i., the cultures were collected and stored in aliquots at −80 °C. Virus titrations were performed in triplicate in Vero cells. 

### 2.7. Immunohistochemistry Assay

For immunohistochemistry, Vero cells grown in 24-well plates were infected with PEDV at an MOI of 0.1 for 1 h at 37 °C. Virus inoculum was then discarded and replaced by a normal growth medium. At 18 h. p. i., the cells were fixed with 4% paraformaldehyde for 15 min, permeabilized with 0.2% Triton X-100 in PBS for 15 min, washed with PBS, air dried, and incubated with rabbit anti-ORF3 (P71-3) polyclonal antibody or mouse anti-PEDV N monoclonal antibody (3F.12) at a dilution of 1:50 in a humidified chamber at 37 °C for 60 min. After washing thrice with PBST (PBS containing 0.1% Tween-20), the cells were incubated for 50 min at 37 °C with the corresponding HRP-labeled goat anti-rabbit IgG or goat anti-mouse IgG at a dilution of 1:200 in PBST. The cells were again washed 3 times with PBST, followed by incubation for 4–5 min at room temperature in diaminobenzidine solution (Solarbio, Beijing, China). Cell staining was examined using a light microscope.

### 2.8. Live Cell Counting and Cell Viability Determination

Vero cells were seeded with 2 × 10^5^ cells/well in 6-well plates, grown to 80% confluence, and washed twice with serum-free medium. They were then infected in triplicate at an MOI of 0.1 with recombinant PEDVs or DR13^att^. After incubation at 37 °C for 1 h, the cells were washed 2 times with PBS and fed with maintenance medium and incubated at 37 °C with 5% CO_2_. At 24 and 36 h.p.i., the supernatants were discarded and the cells were washed twice with PBS, digested for 1 min with trypsin, and resuspended in 0.5 mL PBS for cell counting using a Cellometer (Nexcelom Bioscience, Lawrence, MA, USA).

Cell viability was evaluated using a standard cell counting kit (Kit-8, CCK-8, Beyotime Biotech, Shanghai, China) assay in a 96-well plate according to the manufacturer’s instructions. The CCK-8 allows for very convenient viability analysis by utilizing the live cell membrane permeable tetrazolium salt WST-8 [2-(2-methoxy-4-nitrophenyl)-3-(4-nitrophenyl)-5-(2,4-disulfophenyl)-2H-tetrazolium, monosodium salt], which produced a water-soluble formazan dye upon reduction in live cells. The amount of dye produced was proportional to the number of viable cells in the test system. Briefly, Vero cells (5000 cells/well) were allocated to 96-well tissue culture plates and incubated in 100 µL of DMEM containing 2% heat-inactivated FBS at 37 °C. The next day cells were infected with recombinant PEDVs or DR13^att^ at an MOI of 0.1 in 100 µL DMEM, as described above. At 12, 24, 36, and 48 h.p.i., 10 µL of CCK-8 solution was added to each well and the incubation was continued for another 3 h after which the absorbance of each well was measured at 450 nm. The relative cell viability (%) was calculated and shown as the ratio of the absorbance in infected cells to that in corresponding control cells (vehicle).

### 2.9. Flow Cytometry

Cellular apoptosis was measured in PEDV infected cells by flow cytometry. Briefly, Vero cells were infected with the viruses at MOI of 2 or mock-infected. At 24 h.p.i., the cells were collected, and the surface S protein was stained with a rabbit anti-PEDV S polyclonal antibody (1:200) for 1 h at room temperature (RT) under nonpermeabilized condition. After being washed twice with cell staining buffer (PBS containing 0.1% BSA), cells were subsequently stained with PE-conjugated monkey anti-rabbit secondary antibody (1:200, eBioscience, Catalog No. 12-4739-81) at 4 °C for 30 min. Then, cells were stained using a FITC Annexin V Apoptosis Detection Kit with 7-AAD (1:50 in binding buffer, catalog number 640922, BioLegend, San Diego, CA) according to the manufacturer’s protocol. To exclude PEDV negative cells, the PEDV S protein positive cells were gated and the percentage of apoptotic cells was quantified by a BD FACSCanto II flow cytometer (BD Biosciences, San Jose, CA, USA) following the manufacturer’s instructions. With the kit, cells only positive with Annexin V were classified as early apoptotic and cells only positive with 7-AAD were classified as necrotic. Cells with the above double labeling were categorized as late apoptotic. All the data were analyzed using FlowJo software (version 10.0, Tree Star, Ashland, OR, USA). 

For intracellular staining of cleaved caspase-3, Vero cells infected by PEDV as above were paraformaldehyde-fixed at 24 h post-infection, methanol-permeabilized, and washed with cell staining buffer (PBS containing 0.1% BSA). Cells were then incubated with mouse anti-PEDV N monoclonal antibody (1:200, BioNote, Catalog No. 9191) and rabbit anti-cleaved caspase-3 monoclonal antibody (1:500, Cell Signaling Technology, Catalog No. 9664) for 1 h at RT. Cells were then washed twice using cell staining buffer, stained with PE-conjugated monkey anti-rabbit secondary antibody (1:200, eBioscience, Catalog No. 12-4739-81) and FITC-conjugated rat anti-mouse secondary antibody (1:100, eBioscience, Catalog No. 11-4011-85) for 1 h at RT, and washed again twice with cell staining buffer prior to analysis by flow cytometry. To exclude the PEDV negative cells, the PEDV N protein-positive cell populations were gated and the percentages of cleaved caspase-3 positive cells were quantified by a BD FACSCanto II flow cytometer. All the data were analyzed with FlowJo software (version 10.0, Tree Star, Ashland, OR, USA). 

### 2.10. TUNEL Labeling Assay

Vero cells were grown in 24-well plates on microscope coverslips and infected with PEDVs (rDR13^att^-ORF3^wt^, rDR13^att^-∆ORF3 and DR13^att^) at an MOI of 3. At 8, 16, and 24 hours post-infection, the virus-infected cells were fixed with 4% paraformaldehyde for 15 min at 37 °C and permeabilized with 0.2% Triton X-100 in PBS at RT for 15 min. For apoptotic analysis of PEDV infected cells, the TUNEL (terminal deoxynucleotidyl transferase-catalyzed deoxyuridine phosphate-nick end labeling) assay was performed using a TUNEL BrightGreen Apoptosis Detection Kit (Vazyme Biotech Co. Ltd., Nanjing, China) according to the manufacturer’s instructions. Briefly, Vero cells were rinsed twice with PBS, incubated with rabbit anti-PEDV S polyclonal antibody at 37 °C for 1 h. After washing 3 times in PBS, cells were immersed in TUNEL reaction mixture with Alexa Fluor 594 conjugate goat anti-rabbit IgG (Catalog No. R37117, 1:30) and incubated for 60 min at 37 °C in the dark. After washing 3 times in PBS, the cell nuclei were stained with 4’,6-diamidino-2-phenylindole (DAPI) (1:1000 dilution) at 37 °C for 15 min, after which the cells were mounted on microscope glass slides with FluorSave™ Reagent (Merck Millipore, Billerica, MA, USA, Catalog No. 345789) and visualized under a fluorescent microscope (Axio Scope A1, Carl Zeiss, Germany).

### 2.11. SDS-PAGE and Western Blotting

Vero cells were grown in 6-well plates for 1 day and were mock-infected or infected with PEDV at an MOI of 2. Cells were harvested at 24 h.p.i., and lyzed with 100 μL of ice-cold RIPA buffer (50 mM Tris-HCl, pH 7.4, 150 mM NaCl, 1% Triton X-100, 0.5% sodium deoxycholate, 0.1% SDS, 1 mM EDTA) containing protease and phosphatase inhibitor cocktail (TransGen Biotech Inc., Beijing, China) for 30 min on ice, and clarified by centrifugation at 15,000× *g* for 20 min at 4 °C. The supernatant was collected, and a total protein quantitation was performed using a BCA protein assay (Pierce, Rockford, IL, USA). A 50 µL aliquot of the supernatant was mixed with an equal volume of 2× SDS-PAGE sample buffer and boiled for 5 min, equal amounts of total protein (30 μg) were loaded into each lane, the proteins were separated by electrophoresis in a 12% polyacrylamide gel and transferred onto a PVDF membrane (Pall, New York, NY, USA). Membranes were then blocked with 5% non-fat powdered milk dissolved in TBS (10 mM Tris–HCl (pH 8.0), 150 mM NaCl) containing 0.1% Tween-20 for 1 h at RT and incubated either with rabbit anti-cleaved caspase-3 monoclonal antibody (1:1000, Cell Signaling Technology, Catalog No. 9664), rabbit anti-caspase-3 monoclonal antibody (1:1000, Cell Signaling Technology, Catalog No. 9665), rabbit anti-GAPDH polyclonal antibody (1:1000, Shanghai Sangon Biotech, Catalog No. D110016), or rabbit anti-PEDV S polyclonal antibody in TBS containing 0.1% Tween-20 at 4 °C overnight. After washing with TBS containing 0.1% Tween-20, the membranes were further incubated with the secondary HRP-labeled goat anti-rabbit IgG antibody at a dilution of 1:10,000 for 1 h at RT. The immune-labeled proteins were visualized using ECL Western Blotting Substrate (Pierce, Rockford, IL, USA), according to the manufacturer’s instructions. Images of blots were captured on a chemiluminescence CCD imaging system (ChemiScope 6200T imager, Clinx Science Instruments Co. Ltd., Shanghai, China). To quantify the viral protein production, band densities of target proteins were quantitatively analyzed using the ImageJ software package (https://imagej.nih.gov/ij/) based on the density value relative to the GAPDH protein. 

### 2.12. Statistical Analysis

Determination of growth curves, cell counting, and the cell viability and apoptosis assays were each repeated 3 times, and all the results were presented as the mean ± SD. Statistical analysis was carried out using SPSS 17.0. Differences with a *P* value < 0.05 and < 0.01 were considered to be significant and highly significant, respectively. 

## 3. Results

### 3.1. Generation of Recombinant PEDVs With Different ORF3

**Figure 1:**
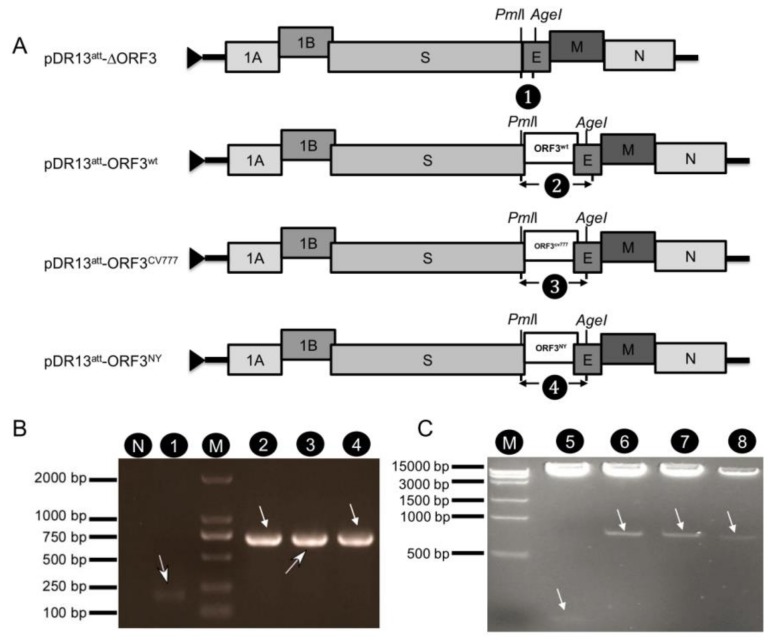
Construction and verification of transfer vectors pDR13^att^-∆ORF3, pDR13^att^-ORF3^wt^, pDR13^att^-ORF3^CV777,^ and pDR13^att^-ORF3^NY^. (**A**). Schematic map of constructed pDR13^att^-∆ORF3, pDR13^att^-ORF3^wt^, pDR13^att^-ORF3^CV777^, and pDR13^att^-ORF3^NY^ transfer vectors. (**B**). PCR was performed across the ORF3 region (primers 1/3R) using the constructed plasmids as templates, and products were analyzed by gel electrophoresis. The numbers refer to the PCR products as indicated in the genome maps in (**A**). Lane N: negative control; lane M: DL 2000 DNA ladder. For primer sequences, see Table 1. (**C**). Electrophoretic verification of the constructed recombinant plasmids by *Pml*I and *Age*I double digestion (2% agarose gel). Lane M: Trans 15K DNA ladder (TransGen Biotech Inc., Beijing, China); lanes 5–8 correspond to plasmids numbered 1–4 in (**B**). Arrows point at the relevant bands the predicted sizes of which are 191 nt and 725 nt for lanes 1 and 2–4 in B and 130 nt and 770 nt for lanes 5 and 6–8 in C, respectively.

We used an earlier established reverse genetics system based on homologous RNA recombination [20] for generating PEDVs expressing different ORF3. To this end, four transfer vectors were constructed for use as templates for the transcription of the different recombination donor RNAs: pDR13^att^-ORF3^wt^, pDR13^att^-ORF3^CV777^, pDR13^att^-ORF3^NY^, and pDR13^att^-∆ORF3. They were constructed by substituting in vector pPEDV-DR13^att^ the ORF3 sequence by the ORF3 sequences of PEDV strains DR13^wt^, CV777 and NY or by entirely deleting the ORF3 sequence from the construct (Figure 1A). The different ORF3 sequences were validated by sequencing and the vector constructs were validated by PCR analysis of the inserts (Figure 1B) and by double restriction digestion analysis with *Pml*I and *Age*I (Figure 1C). 

**Figure 2:**
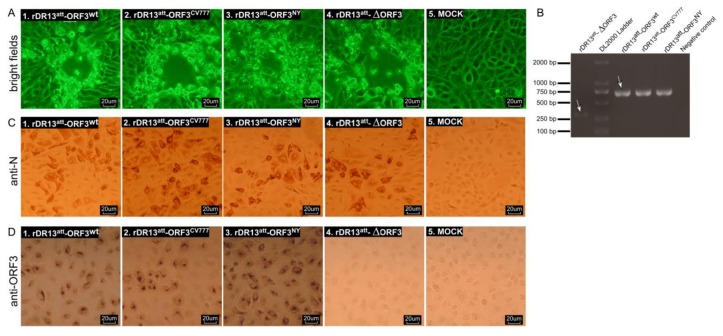
Characterization of recombinant PEDVs. (**A**). Cytopathic effect (CPE) induced by rPEDVs in Vero cells at 40 hours after inoculation with mPEDV-infected LR7 cells that had been transfected with different transfer vectors. Panels 1–4 showed the CPE caused by rDR13^att^-ORF3^NY^, rDR13^att^-ORF3^CV777^, rDR13^att^-ORF3^wt^, and rDR13^att^-∆ORF3, respectively. Images were acquired with a microscope using the LAS AF software. (**B**). To verify the ORF3 region of the recombinant viruses, RT-PCR was performed to amplify the relevant genome fragment with primers mapping in the S gene 3’-end and E gene 5’-end using RNA templates isolated from culture media of Vero cells for rescuing viruses rDR13^att^-ORF3^wt^, rDR13^att^-ORF3^CV777^, rDR13^att^-ORF3^NY^, rDR13^att^-∆ORF3, or negative control. The expected sizes of the RT-PCR products are indicated by arrows. For primer sequences, see Table 1; (**C**) and (**D**). IHC analysis of N protein and ORF3 protein synthesis in Vero cells infected with recombinant PEDVs. Panels 1–4 showed infections with rDR13^att^-ORF3^wt^, rDR13^att^-ORF3^CV777^, rDR13^att^-ORF3^NY^, and rDR13^att^-∆ORF3, respectively. At 18 h. p. i., cells were fixed with 4% formaldehyde and processed for immunohistochemistry using mouse anti-PEDV N monoclonal antibody (3F.12, 1:200) or rabbit anti-ORF3 polyclonal antibody (P71-3, 1:50) as primary antibodies, and the HRP-labeled goat anti-mouse IgG or goat anti-rabbit IgG as second antibodies, respectively. Images were acquired with a microscope using the LAS AF software.

To generate the different rPEDVs, in vitro RNA transcripts from pDR13^att^-ORF3^wt^, pDR13^att^-ORF3^CV777^, pDR13^att^-ORF3^NY^, or pDR13^att^-∆ORF3 were electro-transfected into LR7 cells that had been infected with mPEDV, after which the electroporated cells were co-cultured with Vero cells. CPE appeared in the Vero cells around 40 h post electroporation (Figure 2A). The recombinant viruses rDR13^att^-ORF3^wt^, rDR13^att^-ORF3^CV777^, rDR13^att^-ORF3^NY^, and rDR13^att^-∆ORF3 were purified on Vero cells by terminal dilution and stocks were prepared. For the verification of the recombinant viruses, RT-PCR was performed to amplify specific segments of the RNA genome for sequencing. Using primers mapping in the 3′-end of the S gene and the 5′-end of the E gene, DNA fragments of expected sizes were amplified, i.e., a 725 bp fragment amplified from rDR13^att^-ORF3^wt^, rDR13^att^-ORF3^CV777^, and rDR13^att^-ORF3^NY^, respectively, and a 309 bp fragment amplified from rDR13^att^-∆ORF3 (Figure 2B). Sequencing of the fragments further confirmed the results. Immunohistochemical analysis (IHA) with mouse anti-PEDV N monoclonal antibody showed all the rescued recombinant porcine epidemic diarrhea viruses (PEDVs) to be able to infect the Vero cells (Figure 2C). The IHA analysis also showed ORF3 protein staining with the P71-3 antibody in Vero cells infected with recombinant viruses rDR13^att^-ORF3^wt^, rDR13^att^-ORF3^CV777^, and rDR13^att^-ORF3^NY^, but not in rDR13^att^-∆ORF3 infected and mock-infected cells (Figure 2D). These results confirmed that the intended recombinant viruses rDR13^att^-ORF3^wt^, rDR13^att^-ORF3^CV777^, rDR13^att^-ORF3^NY^, and rDR13^att^-∆ORF3 had been successfully rescued. 

### 3.2. ORF3 Protein Contributes to Virus Proliferation

**Figure 3:**
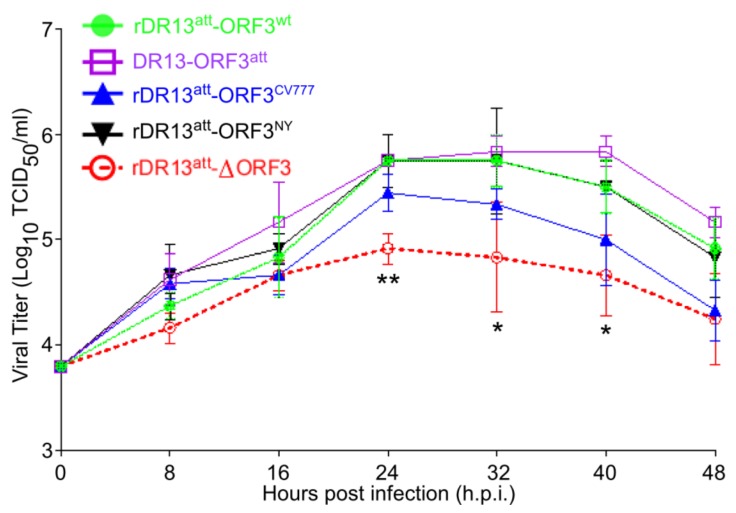
Multi-step growth kinetics of rPEDVs and PEDV DR13^att^ in Vero cells. Vero cells were infected with rDR13^att^-ORF3^wt^, rDR13^att^-ORF3^CV777^, rDR13^att^-ORF3^NY^, rDR13^att^-∆ORF3, and DR13^att^ at a MOI of 0.1. At the indicated times post-infection, cells and culture media were harvested by three rounds of freezing and thawing, followed by centrifugation to remove cell debris. Supernatants were collected and used to measure viral titers. Results were expressed as the mean values from three parallel tests and error bars represent standard deviations (SD). Symbols * and ** indicate the difference between rDR13^att^-∆ORF3 and the other viruses was significant (*p* < 0.05) or very significant (*p* < 0.01), respectively.

We subsequently examined the replication kinetics of the rPEDVs as well as of PEDV DR13^att^ in Vero cells. As shown in Figure 3 (for primary data see Table S1), while their growth kinetics were all quite similar, all ORF3-expressing viruses—including the one expressing ORF3^trun^—grew to higher titers than the virus without ORF3. The three rPEDVs carrying a wild type ORF3-rDR13^att^-ORF3^wt^, rDR13^att^-ORF3^CV777^, and rDR13^att^-ORF3^NY^ reached a maximal titer of approximately 10^5.7^ TCID_50_/mL at ~24–32 h.p.i. The rDR13^att^-∆ORF3 grew less efficiently throughout the testing process, reaching a peak titer of 10^5.3^ TCID_50_/mL, which was approximately 90% compared to the viruses having wild type ORF3. The DR13^att^ vaccine strain reached a titer of 10^5.8^ TCID_50_/mL at 40 h.p.i., the highest titer among the five PEDVs examined. Statistical analysis indicated the difference between the peak titers of rPEDVs with an intact ORF3 and that of the rPEDV without ORF3 to be significant (*p* < 0.05). Because all these PEDVs had the same genetic backbone except for their ORF3, the results indicated that the presence of an intact ORF3 protein-enhanced PEDV proliferation. In addition, also the naturally truncated ORF3 protein as it occurred in strain DR13^att^ exhibited this enhancing effect on virus proliferation. 

### 3.3. ORF3 Protein Contributes to Cell Viability

**Figure 4:**
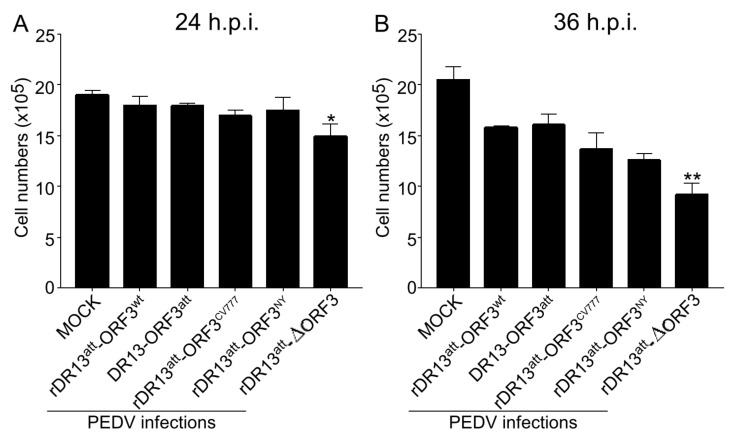
Viable Vero cell counts after infection with rPEDVs and DR13^att^. Vero cells were mock-infected or infected with rDR13^att^-ORF3^wt^, rDR13^att^-ORF3^CV777^, rDR13^att^-ORF3^NY^, rDR13^att^-∆ORF3, and DR13^att^ at a MOI of 0.1. The number of live cells was counted at 24 h.p.i. (in **A**) and 36 h.p.i. (in **B**). Data are represented as the mean ± SD from three parallel tests. Symbols * and ** indicate the difference between rDR13^att^-∆ORF3 and the other viruses is significant (*p* < 0.05) or very significant (*p* < 0.01), respectively.

**Figure 5:**
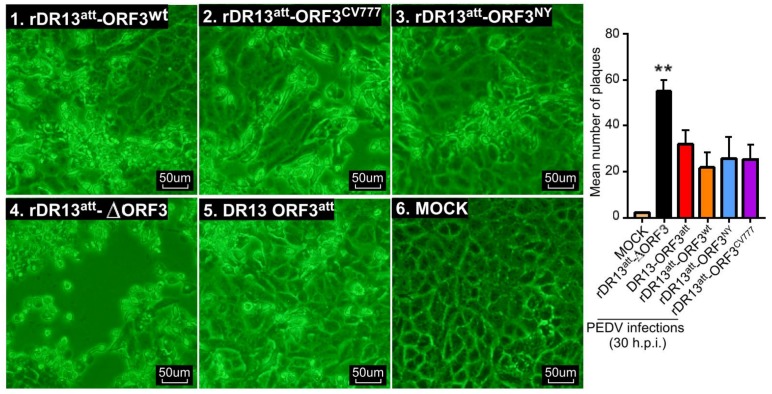
Comparison of cytopathic effect (CPE) caused by different viruses. Vero cells were mock-infected or infected with rDR13^att^-ORF3^wt^, rDR13^att^-ORF3^CV777^, rDR13^att^-ORF3^NY^, rDR13^att^-∆ORF3, and DR13^att^ at a MOI of 0.1. CPE was monitored and photographed at 30 h.p.i. using an inverted microscope at a magnification of 200×. Results shown in the histogram (right panel) represent the mean ± SD from three parallel tests. Symbol ** indicates that the difference of extent of CPE caused between rDR13^att^-∆ORF3 and the other viruses is very significant (*p* < 0.01). Scale bar represents 50 μm.

As more CPE was observed in Vero cells infected with rDR13^att^-∆ORF3, we sought to quantitate the effect of the ORF3 protein on cell viability of the infected cells. To this end, the four rPEDVs and the vaccine strain DR13^att^ were applied to Vero cells at an MOI of 0.1 as described above. First, the live cells were counted at 24 and 36 h.p.i., which showed that their number in cultures infected with rDR13^att^-∆ORF3 was significantly less than when infected with the strains carrying the wild type or truncated ORF3, at both time points (*p* < 0.05, *n* = 3; Figure 4). Pictures taken at 30 h.p.i. also confirmed visually that the extent of CPE caused by infection with rDR13^att^-∆ORF3 was higher than after infection with the other viruses (Figure 5).

**Figure 6:**
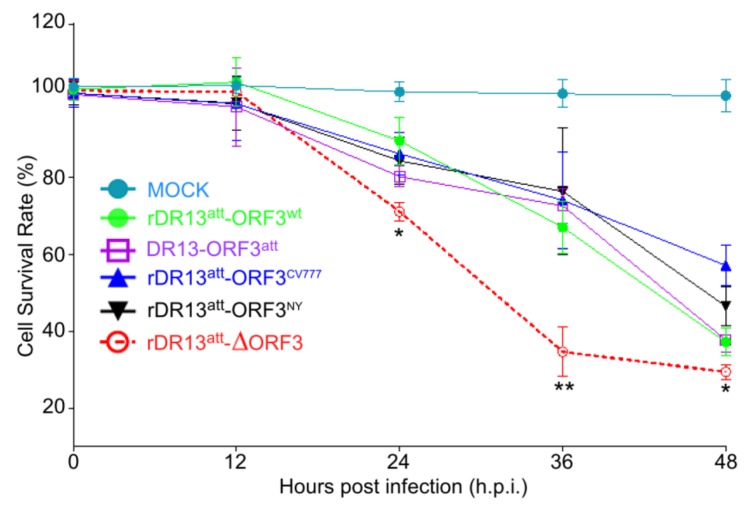
Cell viability after infection with rPEDVs and DR13^att^. Vero cells were mock-infected or infected with rDR13^att^-ORF3^wt^, rDR13^att^-ORF3^CV777^, rDR13^att^-ORF3^NY^, rDR13^att^-∆ORF3, or DR13^att^ at a MOI of 0.1. Cell viability was determined at 12, 24, 36, and 48 h.p.i. and the viability of mock-infected cells was designated as 100%. Results are expressed as a percentage of control ± SD from at least three parallel tests. Symbols * and ** indicate that the difference between the survival rates of cells infected by rDR13^att^-∆ORF3 and other viruses is significant (*p* < 0.05) or very significant (*p* < 0.01), respectively.

To further confirm the effect of ORF3 protein on the condition of infected Vero cells, cellular viability was measured (in fact involving the measurement of formazan dye generated by dehydrogenases in live cells) at different time points after infection with the PEDVs. As indicated in Figure 6, as compared with mock-infected cells, the viability of the virus-infected cells decreased significantly in the course of infection. While the viabilities of the cells infected with the five PEDVs were the same in the early stage of infection (12 h.p.i.), differences appeared at later stages. The cell viabilities observed for rDR13^att^-∆ORF3 at 24, 36, and 48 h.p.i. were all significantly lower than those for the other four PEDVs (*p* < 0.05 at 24, 48 h.p.i.; *p* < 0.01 at 36 h.p.i.). These results further confirmed that ORF3 protein had a beneficial effect on the viability of PEDV infected cells. 

### 3.4. ORF3 Protein Inhibits Apoptosis Induced by PEDVs

**Figure 7:**
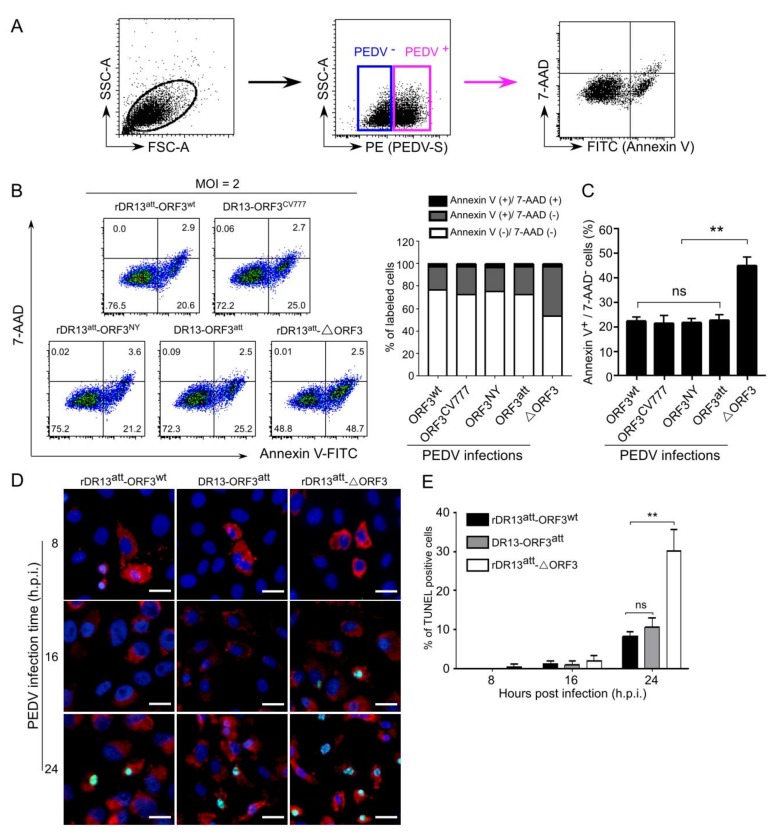
ORF3 has anti-apoptotic effects on PEDV-infected cells. (**A**). Gating strategy to exclude PEDV negative cells before measuring the apoptotic parameters. (**B**). Flow cytometric quantitation of PEDV induced apoptosis. Vero cells cultured in 6-well plates were mock-infected or infected with the indicated viruses at an MOI of 2. At 24 h.p.i., cells were surface-stained using an antibody to the S protein under a non-permeabilized condition and subsequently dually labeled with Annexin V-FITC/7AAD. The percentage of apoptotic cells was quantified in a population of PEDV S protein-positive cells by flow cytometry. The lower left quadrants represent live cells (Annexin V negative/7AAD negative); the lower right quadrants represent early apoptotic cells (Annexin V positive/7AAD negative); the upper right quadrants indicate late apoptotic cells (Annexin V positive/7AAD positive); the upper left quadrants indicate necrotic cells (Annexin V negative/7AAD positive). The figure is representative of three parallel tests. The right graph represents the percentage of cells in each quadrant infected with different viruses. (**C**). Graph summarizing early apoptosis in cells infected with the indicated viruses (*n* ≥ 3; **, *p* < 0.01). (**D**). TUNEL staining of Vero cells after PEDV infection. Infected cells fixed at the indicated time post-infection were stained with an anti-PEDV-S antibody (red), labeled with the TUNEL reagent (green) and nuclei were counterstained with DAPI (blue). Staining was observed with a fluorescent microscope (Axio Scope A1, Carl Zeiss, Germany) and shown as merged pictures. Scale bar represents 50 μm. (**E**). Quantification of TUNEL stained cells. The number of cells with TUNEL positive signals among PEDV infected cells was quantified by counting at least five fields per slide. Results shown in the histogram represent the mean ± SD from three parallel tests. Symbol ** indicates that the difference between the apoptotic parameters of cells infected by rDR13^att^-∆ORF3 and the other viruses is very significant (*p* < 0.01); ns means: not significant.

One possible mechanism by which the above observations might be explained is by the ORF3 protein exerting an inhibitory effect on cell apoptosis. To study this hypothesis, Vero cells were infected with the viruses rDR13^att^-ORF3^wt^, rDR13^att^-ORF3^CV777^, rDR13^att^-ORF3^NY^, rDR13^att^-∆ORF3, and DR13^att^ at an MOI of 2 for apoptotic analysis by flow cytometry at 24 h.p.i. (Figure 7A–C). PEDV infected cells were selected on the basis of S protein expression (Figure 7A) and the percentages of apoptotic cells in the infected cell populations were analyzed by dual Annexin V-7AAD cell labeling (Figure 7B). We observed a significantly larger (*p <* 0.01) early apoptotic fraction in the cells infected with rDR13^att^-∆ORF3 than after infection with the other viruses (Figure 7B,C). No significant difference was observed between DR13^att^ infected cells and cells infected with the full-length ORF3 expressing viruses. This result indicated that full-length ORF3 protein and its C-terminally truncated form may have the same anti-apoptotic effects. To directly observe the different apoptotic states of the cells infected with the PEDVs, a TUNEL assay was carried out following an earlier used method [21]. While no apparent TUNEL-labelling was detected in the PEDV S protein-positive cells at 8 h.p.i., the TUNEL signal was obvious in these infected cells at 16 h.p.i. and even more so at 24 h.p.i. (Figure 7D,E). Whereas the extent of TUNEL labeling in cells infected by rDR13^att^-ORF3^wt^ and DR13^att^ was similar, the TUNEL signal in the cells infected with rDR13^att^-∆ORF3 was significantly higher, particularly at the later time point (*p* *<* 0.01).

### 3.5. ORF3-Mediated Inhibition of Caspase-3 Cleavage in PEDV Infected Cells

**Figure 8:**
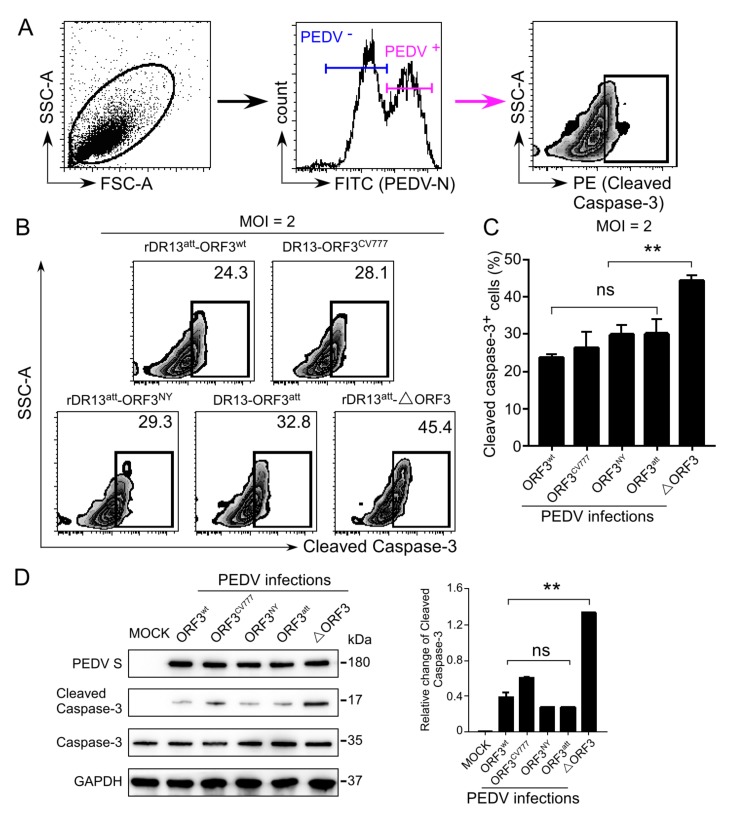
Detection of cleaved caspase-3 by flow cytometry and Western blot in PEDV infected Vero cells. **(A**). Gating strategy to enrich for infected cells by selection on the basis of N protein detection before measuring cleaved caspase-3 by flow cytometry. (**B**). Representative flow cytometry plots indicating intracellular cleaved caspase-3 levels in Vero cells infected by rDR13^att^-ORF3^wt^, rDR13^att^-ORF3^CV777^, rDR13^att^-ORF3^NY^, DR13^att^, and rDR13^att^-∆ORF3. Vero cells cultured in 6-well plates were mock-infected or infected with the viruses at an MOI of 2 for 24 h. Cells were then fixed, methanol permeabilized, and stained using rabbit anti-cleaved caspase-3 specific antibody and mouse anti-PEDV N monoclonal antibody as primary antibodies and PE-conjugated monkey anti-rabbit IgG and FITC-conjugated rat anti-mouse IgG as secondary antibodies, respectively. The percentage of cleaved caspase-3 expressing cells was quantified by flow cytometry in PEDV N protein-positive cell populations. The figure is representative of three parallel tests. (**C**). Comparative analysis of the levels of cleaved caspase-3 induced by the different viruses using flow cytometry (n ≥ 3; **, *p* < 0.01). (**D**). Western blot analysis of caspase-3 cleavage in infected cells. Vero cells were infected with the viruses at an MOI of 2. At 24 h.p.i., cells were harvested and lysed after which proteins were separated by PAGE and immunoblotted using antibodies recognizing PEDV S protein (top panel), cleaved caspase-3 (second panel), and caspase-3 (third panel). The blot was also reacted with rabbit polyclonal antibody against GAPDH to verify equal protein loading (bottom panel). The figures are representative of three parallel experiments. The plot at the right shows the relative levels of cleaved caspase-3 after normalization with GAPDH controls. Error bars represent standard deviations calculated from three replicates (n ≥ 3; **, *p* < 0.01); ns means: Not significant.

One criterium to judge the apoptotic state of cells is by measuring the level of cleavage activation of caspase-3 in those cells [22,23]. Activated caspase-3 has been demonstrated earlier in PEDV-induced apoptosis [24]. To check the cleaved caspase-3 level induced by the PEDV infection, Vero cells were infected with the viruses at an MOI of 2 (Figure 8). PEDV infected cells were gated by excluding the viral N protein negative cells (Figure 8A). The percentage of cells with cleaved caspase-3 was analyzed by flow cytometry (Figure 8B). Consistent with the results from the dual Annexin V/7AAD cell labeling, we found a significantly higher (*p <* 0.01) cleaved caspase-3 level in the cells infected with rDR13^att^-∆ORF3 than when infected with the other viruses (23.3–28.1% vs. 45.4%; Figure 8B,C).

We further verified the caspase-3 cleavage states in PEDV infected Vero cells by Western blot. Again, we observed a significant difference in the level of cleaved caspase-3 in rDR13^att^-∆ORF3 infected cells as compared with cells infected by the other viruses at 24 h.p.i. (Figure 8D). In contrast, when checking at the levels of S protein and uncleaved caspase-3, no obvious differences were found between the differently-infected cells. At the earlier infection time of 12 h.p.i., cleaved caspase-3 could not be detected in any of the infected cell populations (Figure S2). Interestingly, no significant difference was observed between the caspase-3 cleavage levels in DR13^att^ infected cells expressing the truncated ORF3 protein and cells infected with the viruses expressing the full-length ORF3 proteins. These results were consistent with the findings obtained using the Annexin V/7AAD assay, implying that the C-terminally truncated ORF3 protein may have the same anti-apoptotic effects as the full-length form.

## 4. Discussion

Considering ORF3 is the only conserved accessory gene in all coronaviruses, it is safe to assume its encoded protein to be beneficial for these viruses’ natural infection. Yet, there is thus far limited evidence for its precise role. In the case of PEDV, studies about ORF3 protein have been incoherent; many involved gene sequence analyses but few were about its biological functions. In a recent study, PEDV ORF3 protein was reported to have no effect on cell apoptosis [24]. In the present study, using a unique system of reverse genetics, the ORF3 protein was demonstrated to enhance virus proliferation by a mechanism involving inhibition of cell apoptosis. 

To enable a sensitive analysis of its effect on infection, we rescued several PEDVs differing in the nature of their ORF3. All the viruses had the same DR13^att^ genetic backbone, their only difference being their ORF3. Rather than also making a recombinant virus expressing a truncated ORF3 protein (ORF3^trun^), we used the DR13^att^ strain to examine the behavior of ORF3^trun^ protein. We used these viruses to investigate whether the ORF3 protein plays a role in viral proliferation. By comparing the growth kinetics of the different viruses, we found that rPEDVs carrying ORF3, including also the one expressing the truncated form, proliferated faster and to higher titers than the virus without the gene, suggesting the ORF3 protein promotes the proliferation of PEDV in the cell culture system. 

While growing the recombinant viruses, we noticed the development of CPE in cells infected with rDR13^att^-∆ORF3 to be more severe than with viruses carrying intact or truncated ORF3. Consistently, when measuring cellular viability in the course of infection by these viruses, we observed the same trend, i.e., enhanced cell death in the absence of ORF3 protein. This led us to suppose that the ORF3 protein, whether intact or truncated, reduces cell apoptosis, considering that infection by PEDV has been shown to induce this cellular reaction [21,25]. Consistent with this hypothesis, we found both ORF3^wt^ and ORF3^trun^ protein to exhibit anti-apoptotic properties. 

Apoptosis is considered to be a host innate defense mechanism that disrupts viral replication by eliminating virus-infected cells. Viruses have consequently developed strategies to reduce apoptosis, which, in turn, prevents premature cell death, thereby maximizing progeny virus production [26]. In this study, we observed the use of such a strategy by PEDV. The viruses carrying an ORF3 not only grew to higher titers than the virus lacking the ORF, but their infection also caused less apoptosis, as judged by apoptotic indicators such as TUNEL labeling, translocation of phosphatidylserine from the inner to the outer leaflet of the plasma membrane and activation of caspase-3. The observations indicate inhibition of procaspase-3 activation to be the possible mechanism of ORF3 protein-mediated host cell apoptosis. On the other hand, many viruses have the ability to actively induce apoptosis to facilitate the release and dissemination of viral progeny to neighboring cells. This pro-apoptotic property is one of the cytolytic effects of viral infections causing CPE in vitro, and it supposedly plays a pathogenic role by contributing to cell damage, tissue injury, and disease severity in vivo. This process has been observed particularly in RNA viruses, including *Coronaviridae* [27,28]. In addition, for PEDV, the induction of apoptotic cell death has recently been demonstrated *in vitro* and *in vivo*. The process was found to be caspase-independent but to be mediated by activation of the mitochondrial apoptosis-inducing factor [21]. While apoptosis facilitates the release of virions from infected cells, proper timing of its induction seems desirable for the virus, as a too early breakdown of the cells would reduce the time and space for its propagation. Hence, we propose that, while PEDV apparently takes advantage of the effects of cellular apoptosis, its ORF3 protein may function to delay these processes in order to extend time and space for the virus to multiply. This may be a built-in mechanism of PEDV to maximize the production of its offspring. 

While truncation of the ORF3 protein was found by Wang et al. to strongly reduce the protein’s ion channel activity [16], in our study it did not influence the protein’s effects on virus proliferation and cell apoptosis. Since the same or similar truncations have been found repeatedly in several independently obtained PEDV strains after serial passaging, there is an apparent selection for viruses still expressing the N-terminal part of the protein. It is tempting to speculate that this selection aims to maintain the favorable growth phenotype of the virus. Loss of the downstream C-terminal part of the protein is associated with attenuation of virulence in the natural host, suggesting that, as earlier proposed by Wang et al. [16], the ORF3 protein’s ion channel activity is associated with the virulence of PEDV. Other functions of this C-terminal part, however, such as interference with innate host immunity [29], cannot be excluded and this warrants further research. 

Earlier studies on the function of the PEDV ORF3 protein have not yielded a consistent picture. Wang et al. used a siRNA approach targeting the viral sub-genomic mRNA3, which encodes the ORF3 protein to study its role in viral replication. While they did observe a strong reduction in infectious virus production for the wild type CV777 strain, no effect was detected for its attenuated derivative, which carried an ORF3^trun^ [16]. Though the actual effect of the siRNA treatment on mRNA3 or ORF3 protein levels was not assessed, their results were consistent with a positive role in virus production for the wild type but not for the truncated ORF3 protein. In a study by Ye et al. a Vero cell line stably expressing the wild type ORF3 protein was established [17]. Virus yields in these cells were found to be similar to those in the parental cells when infected with field strain CH/YNKM-8/2013. A clear increase in infectious virus production was, however, observed with attenuated PEDV strains CV777 and AH-M. Quite a mysterious ORF3 protein was described by Jongkaewwattana’s laboratory [13,30]. This intact ORF3 protein, named ORF3_NP12_ and derived from the Chinese strain JS-2004-2, was found to block the rescue of the infectious virus when its gene was incorporated into strain PEDV_AVCT12_. The rescue was, however, successful when the full-size gene was replaced by its truncated form. The inhibitory effect of ORF3_NP12_ on replication, which was even observed in the context of infection by a totally different virus (porcine reproductive and respiratory syndrome virus), was attributed to two residues (F81 and M167) which, when mutated (F81L or M167S), restored the normal phenotype. It remains puzzling, however, how the strain JS-2004-2 carrying ORF3_NP12_ was able to cope with the inhibitory of effects of this ORF3 protein.

In conclusion, our study demonstrates PEDV ORF3 protein to promote virus proliferation through suppressing cellular apoptosis. However, we cannot exclude other mechanisms by which the ORF3 protein facilitates viral replication. It might, for instance, do so through its direct binding to the viral spike protein [18] or by interacting with cellular proteins [31]. Clearly, more research is needed to learn about the mechanism of the ORF3 protein’s functioning in the proliferation of the virus.

## Figures and Tables

**Table 1:** Primers used for plasmid constructions and recombinant porcine epidemic diarrhea virus (rPEDV) verifications.

Primer	Sequence (5′-3′)	Nucleotides	Location
1	TGAAAAGGTC***CACGTG***CAGTGATGTTTCTTGGACTTTTTCAATACACGAT	24,737–24,786	S-ORF3
2	GGTCAATTCGCATGTAAGACTTATAAACTCTA	25,513–25,544	ORF3
3F	GACTCAATTCAACTAGACGAGTATGCTACAATTAGTGAATGA	25,342–25,383	NCR-E
3R	TCATTCACTAATTGTAGCATACTCGTCTAGTTGAATTGAGTC	25,342–25,383	NCR-E
4	TACGAAGCTTTTGAAAAGGT***CCACGTG***CAGTGAACTCAATTCAACTAGACGAGTATGCTACAATTAGTG	24,726–24,758 and 25,343–25,378	S and E
5	ATAGGTGTGTAAACTGCGCTA	25,480–25,500	E
6	CCGCTAGCGCTACCGGACTCAGAT***CTCGAG***CTCATGTTTCTTGGACTTTTT	24,578–24,775	S-ORF3
7	CGCCATGGTGGCGACCGGT***GGATCC***CGTTCACTAATTGTAGCATACTC	25,360–25,380	NCR-E
8	CGCCATGGTGGCGACCGGT***GGATCC***CGTCATTCACTAATTGTAGCATACTC	25,360–25,383	NCR-E

**Note:** 1. The location of primers is relative to the full genome sequence of the attenuated PEDV DR13 (JQ023162). 2. Homology arm sequences in primers 6 to 8 are in grey. 3. The restriction enzyme sites are marked as italic, bold, and underlined letters. NCR, the non-coding region between the ORF3 and E gene.

## Supplementary Materials

The following data are available online at https://www.mdpi.com/1999-4915/12/2/214/s1. Figure S1: Alignments of nucleotide and amino acid sequences of PEDV ORF3s and their proteins studied in this paper; Figure S2: Western blot analysis of caspase-3 activity at 12 h.p.i.; Table S1: Growth curve values (Log_10_ TCID_50_/mL) of PEDVs at different times post-infection of Vero cells.
