# Supplementary material for: Porcine Epidemic Diarrhea Virus (PEDV) ORF3 Enhances Viral Proliferation by Inhibiting Apoptosis of Infected Cells
Source: Viruses. 2020 Feb 14;12(2):214. doi: 10.3390/v12020214 (PMC7077256; doi:10.3390/v12020214)
Supplement: Supplementary file 1 [file viruses-12-00214-s001.zip › Supplementary Files/Supplemental figures.docx]

**Supplementary figures**


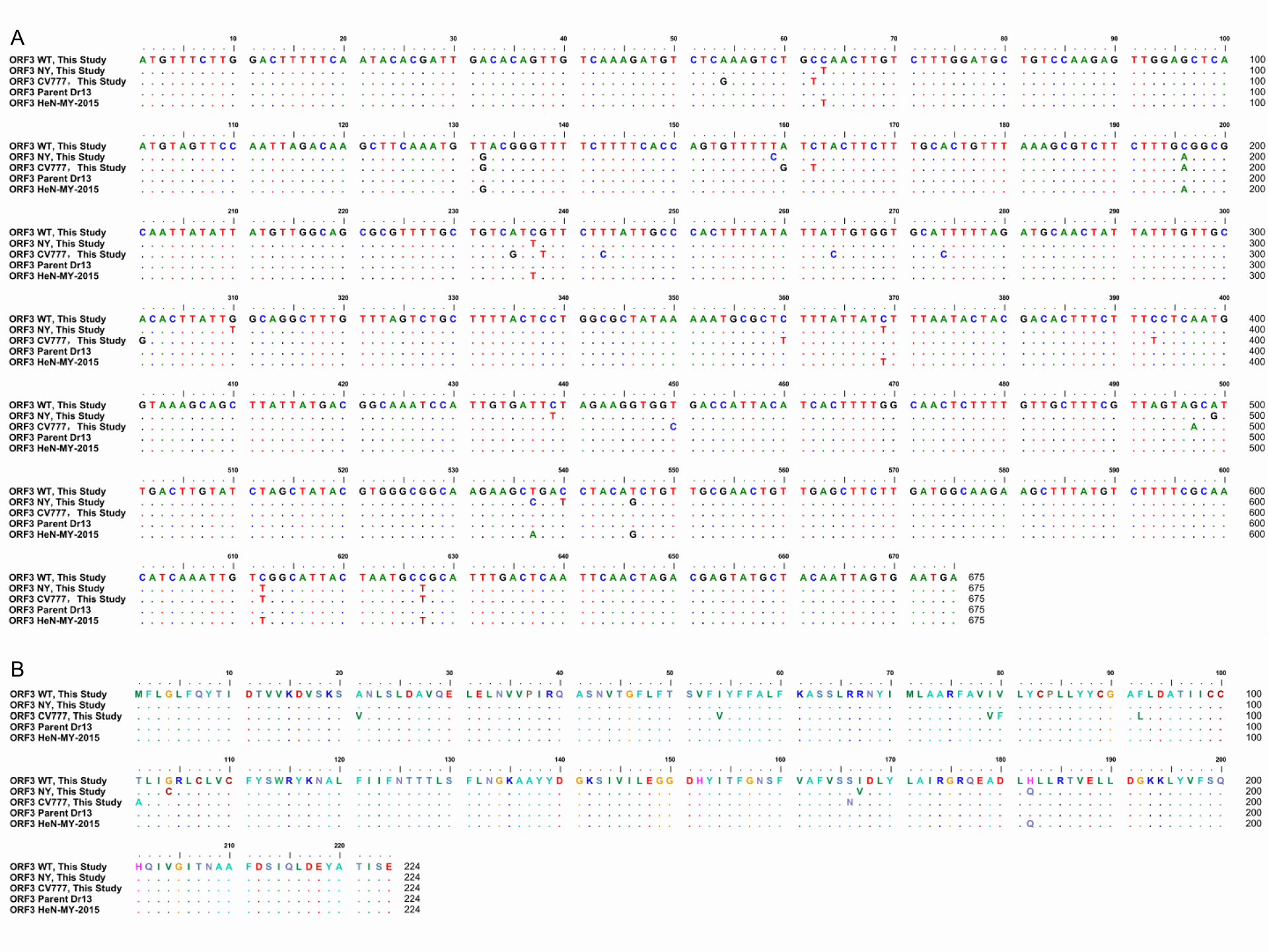


***Figure S1*.** Alignments of nucleotide and amino acid sequences of PEDV ORF3s and their proteins studied in this paper. A. Multiple alignments of nucleotide sequences; B. Multiple alignments of deduced amino acid sequences. The PEDV parent DR13 and a clinical PEDV HeN-MY-2015 strain (GenBank accession no. KU641647) sequences were also included. A dot (.) indicated that this nucleotide in the sequence is identical to the corresponding one in the top sequence. The multiple sequence alignments were performed using the BioEdit software (Version 7.0).


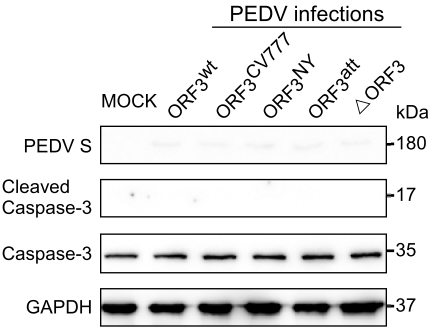


***Figure S2*.** Western blot analysis of caspase-3 activity at 12 h.p.i. Vero cells were infected using the indicated viruses at the MOI of 2, cellular lysates were collected at 12 h.p.i, resolved by SDS-PAGE, transferred to a PVDF membrane, and immunoblotted using an antibody that recognizes PEDV S protein (top panel), cleaved caspase-3 (second panel) or caspase-3 (third panel). The blot was also reacted with rabbit polyclonal antibody against the GAPDH gene to verify equal protein loading (bottom panel).
