# Supplementary material for: Porcine Epidemic Diarrhea Virus (PEDV) ORF3 Enhances Viral Proliferation by Inhibiting Apoptosis of Infected Cells
Source: Viruses. 2020 Feb 14;12(2):214. doi: 10.3390/v12020214 (PMC7077256; doi:10.3390/v12020214)
Supplement: Supplementary file 1 [file viruses-12-00214-s001.zip › Supplementary Files/Supplemental table 1.docx]

**Supplementary tables**

**Table S1**. Growth curve values (Log_10_ TCID_50_/ml) of PEDVs at different times post infection of Vero cells.

| **PEDVs** | **8 h.p.i.** | **16 h.p.i.** | **24 h.p.i.** | **32 h.p.i.** | **40 h.p.i.** | **48 h.p.i.** |
| --- | --- | --- | --- | --- | --- | --- |
| **rDR13^att^-ORF3^wt^** | 4.38 ± 0.07 | 4.83 ± 0.22 | 5.72 ± 0.03 A | 5.75 ± 0.14 A | 5.50 ± 0.14 A | 4.92 ± 0.17 |
| **DR13-ORF3^att^** | 4.63 ± 0.14 | 5.17 ± 0.22 | 5.76 ± 0.01 A | 5.83 ± 0.08 A | 5.83 ± 0.08 A | 5.17 ± 0.08 |
| **rDR13^att^-ORF3^CV777^** | 4.58 ± 0.08 | 4.67 ± 0.11 | 5.44 ± 0.10 A | 5.33 ± 0.08 A | 5.01 ± 0.25 A | 4.33 ± 0.17 |
| **rDR13^att^-ORF3^NY^** | 4.67 ± 0.17 | 4.92 ± 0.08 | 5.75 ± 0.14 A | 5.75 ± 0.29 A | 5.51 ± 0.14 A | 4.83 ± 0.22 |
| **rDR13^att^-∆ORF3** | 4.17 ± 0.08 | 4.67 ± 0.08 | 4.92 ± 0.08 B | 4.83 ± 0.30 B | 4.67 ± 0.22 B | 4.25 ± 0.25 |

**Note:** Letters beside data indicate differences between PEDVs at a specific h.p.i.. B symbolizes significance (*P* < 0.05) as compared with viruses marked A.
